# Relationship between alcohol consumption and telecommuting preference‐practice mismatch during the COVID‐19 pandemic

**DOI:** 10.1002/1348-9585.12331

**Published:** 2022-05-04

**Authors:** Chihiro Watanabe, Yusuke Konno, Ayako Hino, Masako Nagata, Keiji Muramatsu, Seiichiro Tateishi, Mayumi Tsuji, Akira Ogami, Reiji Yoshimura, Yoshihisa Fujino, CORoNaWork project

**Affiliations:** ^1^ Department of Psychiatry University of Occupational and Environmental Health, Japan Kitakyushu Japan; ^2^ Department of Environmental Epidemiology Institute of Industrial Ecological Sciences, University of Occupational and Environmental Health, Japan Kitakyushu Japan; ^3^ Department of Mental Health Institute of Industrial Ecological Sciences, University of Occupational and Environmental Health, Japan Kitakyushu Japan; ^4^ Department of Occupational Health Practice and Management Institute of Industrial Ecological Sciences, University of Occupational and Environmental Health, Japan Kitakyushu Japan; ^5^ Department of Preventive Medicine and Community Health, School of Medicine University of Occupational and Environmental Health, Japan Kitakyushu Japan; ^6^ Department of Occupational Medicine, School of Medicine University of Occupational and Environmental Health, Japan Kitakyushu Japan; ^7^ Department of Environmental Health, School of Medicine University of Occupational and Environmental Health, Japan Kitakyushu Japan; ^8^ Department of Work Systems and Health Institute of Industrial Ecological Sciences, University of Occupational and Environmental Health Kitakyushu Japan

**Keywords:** alcohol consumption, COVID‐19, Japan, occupational health, telecommuting

## Abstract

**Objective:**

This study examined the association between increased alcohol consumption and telecommuting, comparing employees who expressed a preference for telecommuting and those who did not.

**Methods:**

We conducted an internet monitor survey. Responses from 20 395 of the 33 302 participants were included in the final sample. Participants were asked about their desire for and frequency of telecommuting, and about changes in alcohol consumption under the COVID‐19 pandemic. Data were analyzed by logistic regression analysis.

**Results:**

The ratio of increased drinking in those who telecommuted at least once a week was significantly different (OR = 1.29, 95% CI 1.16–1.43, *p* < .001). The ratio of increased drinking in participants for whom telecommuting was not preferred was significantly different (OR = 1.08, 95%CI 1.02–1.14, *p* = .002). Since the interaction term was significant in preliminary analysis, stratification was performed. Participants who telecommuted despite preferring not to do so reported significantly increased alcohol consumption, as revealed by a multivariate analysis (OR = 1.53, 95% CI 1.18–2.00, *p* < .001). Participants who expressed a preference for telecommuting showed no such increase (OR = 1.12, 95% CI 0.98–1.27, *p* = .074).

**Conclusions:**

Under the COVID‐19 pandemic, telecommuting that involves a mismatch with employee preference for way of working may be a new risk factor for problematic drinking.

## 1 INTRODUCTION

With the global outbreak of the new coronavirus, people in many parts of the world are telecommuting. Telecommuting is a way of working that involves working from a location other than one's regular office or place of business, for example, at home or in a co‐working space.^1^ Telecommuting means that one can avoid the flow of people on public transportation and in public spaces during regular commuting, and have fewer contacts with other people at business premises. Therefore, telecommuting is recommended as a measure to combat the spread of the new coronavirus in many countries, including Japan.^2^, ^3^

Mismatches in telecommuting preferences are more likely to occur in the COVID‐19 pandemic than in the past. There are merits and disadvantages to telecommuting.^4^, ^5^, ^6^, ^7^ Merits include increased opportunities to work at one's own pace, working in one's own style, lack of stress of commuting, and reduced travel time. Demerits include difficulties in communication and increased feelings of isolation, lack of space and a suitable desk or chair for work, and difficulty in switching between work and private life. The relative importance of these factors varies among individuals, some of whom will prefer to work from home while others will not. Whereas in the past employees might have had some degree of choice regarding how they worked, today telecommuting may be imposed, regardless of employee preference. This is because telecommuting is recommended as part of the response to the COVID‐19 pandemic, for public health reasons. As a result, employees who feel that the disadvantages of telecommuting outweigh the advantages may have to telecommute against their wishes. This mismatch leads to psychological distress.^8^

A study conducted on Norwegian employees who telecommute more than 15 h per week found that telecommuting may increase alcohol consumption.^9^ It was concluded that in response to the instigation of their new style of working, telecommuters drank more to reduce their psychological distress, which included feelings of anxiety and loneliness. However, the data used in this study are pre‐COVID‐19 pandemic. Therefore, this study does not consider the mismatch that has occurred since the COVID‐19 pandemic, where people telecommute despite not wanting to. According to a survey by Japan's Ministry of Finance, spending on alcoholic beverages by working households in December 2020 was higher than in the previous year, when COVID‐19 was not prevalent.^10^ By contrast, according to the Japan Foodservice Association (JF) Food Service Industry Market Trend Survey, sales of alcoholic beverages in restaurants in December 2020 were lower than in the year before.^11^

These findings suggest that the consumption of alcohol in Japanese households is on the rise, and this phenomenon is likely to include alcohol consumption by telecommuters. In this internet survey‐based study, therefore, we predicted, first, that telecommuting would be associated with increased alcohol consumption in Japan, and second, that this association would be significantly stronger among employees who did not prefer to telecommute.

## 2 METHODS

### 2.1 Study design and participants

This study was done as part of the Collaborative Online Research on the Novel‐Coronavirus and Work (CoroNaWork) Project. This project involved an extensive cross‐sectional study conducted on the internet in Japan from December 22–26, 2020, investigating the health status of employees during the COVID‐19 pandemic. Details of the study protocol are available elsewhere.^12^ Briefly, data were collected from 33 302 workers who were under contract at the time of survey and who agreed to participate. They were assigned to the study by prefecture, occupation, and gender. Of these, 27 036 were included in this study, after excluding those whose responses were judged to be invalid. Invalid responses included those with extremely short response times, those reporting being less than 140 cm tall or less than 30 kg in weight, and inconsistent responses to multiple identical questions. Furthermore, we excluded workers engaged in jobs where telecommuting is not possible, such as on‐site work. Finally, 20 395 workers were included in the analysis. The research was approved by the Ethics Committee of the University of Occupational and Environmental Health, Japan (Reference No. R2‐079). Informed consent was obtained via a form on the website.

### 2.2 Assessment of telecommuting preference and frequency

We used a questionnaire to investigate employees' attitudes toward and frequency of telecommuting. The following single‐item question was used to examine their telecommuting status: “How often do you currently telecommute?” Respondents who answered, "Four or more days a week,” "Two or more days a week,” or “One or more days a week” were categorized as “telecommuting frequency once a week or more;” others were categorized as “telecommuting frequency less than once a week”.

We also used the following single‐item question to determine participants' telecommuting preferences: “Would you like to telecommute?” Respondents who answered, “I would rather work at the regular workplace” or “I would prefer to work at the regular workplace as much as possible” were coded as “I do not prefer to telecommute”. Respondents who answered, “I prefer to work from home as much as possible,” “I rather prefer to work from home,” or “I don't care which,” were classified as “I don't mind telecommuting”.

### 2.3 Assessment of changes in alcohol consumption

We assessed changes in participants’ alcohol consumption during the COVID‐19 pandemic. Those who answered “increased” were classified as “with increase” and others as “without increase.”

### 2.4 Other covariates

The following survey items were considered as potential confounding factors: age, sex, marital status, equivalent income (56–306 JPY/318–469 JPY/465–1050 JPY), educational background (junior high school/ high school/ university, graduate school, vocational school, junior college), smoking, job type (mainly desk work/ mainly work involving communicating with people), number of employees at the regular workplace (1–29/30–99/100–999/≥1000), Kessler 6 scale (K6) score (≥5/5>) and cumulative incidence of COVID‐19 in the prefecture. In addition, the cumulative incidence of COVID‐19 in the prefecture of residence from the time of the survey to 1 month before was used as a community‐level variable. This latter information was collected from the websites of public institutions.

### 2.5 Statistical analysis

Age was used as a continuous variable, while other items were presented as categorical variables, using percentages. We conducted a multilevel logistic model nested in the prefectures of residence, with change in alcohol consumption as the dependent variable and preference for telecommuting and frequency of telecommuting as independent variables. To adjust for potential confounders, adjustment for age, sex, marital status, equivalent income, educational level, smoking, alcohol consumption, job type, number of employees at the workplace, and cumulative incidence rate of COVID‐19 in the prefecture was used as a covariate. We further examined the interaction term between telecommuting frequency and telecommuting preference. Since the interaction term was significant in preliminary analysis, stratification was performed. All statistical analyses were performed using Stata (Stata Statistical Software: Release 16. College Station, TX: StataCorp LLC.). We considered *p* values less than .05 as statistically significant.

## 3 RESULTS

Table 1 presents baseline characteristics of the participants. Of the 20 395 participants whose data were used, 5271 telecommuted at least 1 day per week. The mean age, percentage of married people, and percentage of smokers were similar in the two populations. The proportion of men, the proportion of college graduates, and annual income tended to be higher among those who telecommuted at least 1 day per week.

**TABLE 1:** Characteristics of participants by frequency of telecommuting

	Telecommuting frequency			
	Hardly ever	≧1 day/week		
			Preference of telecommuting	
		All subjects of telecommuting	Prefer to telecommute	Not prefer to telecommute
	*n* = 15 124	*n* = 5271	*n* = 4620	*n* = 651
Age, mean (SD)	46.4 (10.6)	48.8 (10.2)	48.7 (10.2)	49.5 (10.0)
Sex, male	6910 (45.7%)	3054 (57.9%)	2638 (57.1%)	416 (63.9%)
Marriage status, married	8540 (56.5%)	3031 (57.5%)	2619 (56.7%)	412 (63.3%)
Educational background				
Junior high school	137 (0.9%)	36 (0.7%)	34 (0.7%)	2 (0.3%)
High school	3637 (24.0%)	791 (15.0%)	701 (15.2%)	90 (13.8%)
University, graduate school, vocational school, junior college	11350 (75.0%)	4444 (84.3%)	3885 (84.1%)	559 (85.9%)
Equivalent income (million JPY)				
56–306	4697 (31.1%)	1434 (27.2%)	1314 (28.4%)	120 (18.4%)
318–469	5026 (33.2%)	1421 (27.0%)	1242 (26.9%)	179 (27.5%)
475–1050	5401 (35.7%)	2416 (45.8%)	2064 (44.7%)	352 (54.1%)
Job type				
Mainly desk work	9416 (62.3%)	4052 (76.9%)	3607 (78.1%)	445 (68.4%)
Mainly work involving communicating with people	5708 (37.7%)	1219 (23.1%)	1013 (21.9%)	206 (31.6%)
Number of employees in the workplace				
1–29	2732 (18.1%)	1955 (37.1%)	1846 (40.0%)	109 (16.7%)
30–99	4321(28.6%)	658 (12.5%)	569 (12.3%)	89 (13.7%)
100–999	4173 (27.6%)	979(18.6%)	813 (17.6%)	166 (25.5%)
≥1000	3898 (25.8%)	1679 (31.9%)	1392 (30.1%)	287 (44.1%)
Current smoke	3677 (24.3%)	1405 (26.7%)	1217 (26.3%)	188 (28.9%)
Increasing alcohol consumption	1557 (10.3%)	686 (13.0%)	576 (12.5%)	110 (16.9%)
Alcohol consumption frequency				
6–7 days a week	3085 (20.4%)	1246 (23.6%)	1078 (23.3%)	168 (25.8%)
4–5 days a week	1102 (7.3%)	526 (10.0%)	457 (9.9%)	69 (10.6%)
2–3 days a week	1856 (12.3%)	695 (13.2%)	599 (13.0%)	96 (14.7%)
Less than 1 day a week	2605 (17.2%)	925 (17.5%)	794 (17.2%)	131 (20.1%)
Hardly ever	6476 (42.8%)	1879 (35.6%)	1692 (36.6%)	187 (28.7%)

The odds ratios (ORs) of frequency and preference for telecommuting and alcohol consumption estimated by the logistic model are shown in Table 2. The ratio of increased drinking in those who telecommuted at least once a week was significantly different in univariate analysis compared to those that did little to no telecommuting as a baseline (OR = 1.29, 95% CI 1.16–1.43, *p* < .001). Multivariate analysis showed similar results (OR = 1.17, 95%CI1.06–1.31, *p* = .003). The ratio of increased drinking in participants for whom telecommuting was not preferred was significantly different in univariate analysis using the preferred group as a baseline (OR = 1.08, 95% CI 1.02–1.14, *p* = .002). There is no significant difference in multivariate analysis. (OR = 1.03, 95% CI 0.94–1.13, *p* = .486). In the model adding interaction, the interaction was significant. (*p* = .128). The interaction term between frequency of telecommuting and telecommuting preference was significant.

**TABLE 2:** Relationship between frequency or preference of telecommuting and the increasing amount of alcohol consumption

	Univariate				Multivariate^a^			
	OR	95%	CI	*p*	OR	95%	CI	*p*
Frequency of telecommuting								
Telecommuting Frequency less than once a week	Reference				Reference			
Telecommuting frequency once a week or more	1.29	1.16	1.43	<.001	1.17	1.06	1.31	.003
Preference of telecommuting								
Don't mind Telecommuting	Reference				Reference			
No preference of Telecommuting	1.08	1.02	1.14	.002	1.03	0.94	1.13	.486

^a^ Multivariate model adjusted for age, sex, marital status, equivalent income, educational level, smoking, alcohol consumption, job type, K6, number of the employee at the workplace, and cumulative incidence rate of COVID‐19 at the prefecture.

Table 3 shows the odds ratios (ORs) for the frequency of telecommuting and the increase in alcohol consumption for each telecommuting preference category, as estimated by the logistic model. Among participants who did not wish to telecommute, the ratio of increased drinking in the group that telecommuted at least once a week was significantly different in univariate analysis from the group that did little to no telecommuting as a baseline (OR = 1.88, 95% CI 1.49–2.38, *p* < .001). Multivariate analysis showed similar results (OR = 1.62, 95%CI 1.25–2.12, *p* < .001). There was a significant difference in univariate analysis in the percentage of increased drinking in the group who preferred to telecommute at least once a week with the group who rarely telecommuted as baseline (OR = 1.26, 95% CI 1.13–1.41, *p* < .001). However, in multivariate analysis, there was no significant difference (OR = 1.10, 95% CI 0.98–1.26, *p* < .001).

**TABLE 3:** Relationship between frequency of telecommuting and increased alcohol consumption for each telecommuting preference

	Univariate					Multivariate^a^			
	OR	95%		CI	*p*	OR	95%	CI	*p*
Among subjects with no preference of telecommuting									
Telecommuting frequency less than once a week	Reference					Reference			
Telecommuting frequency once a week or more	1.88	1.49	2.38		<.001	1.53	1.18	2.00	<.001
Among subjects with don't mind telecommuting									
Telecommuting frequency less than once a week	Reference					Reference			
Telecommuting frequency once a week or more	1.26	1.13	1.41		<.001	1.12	0.98	1.27	.074

^a^ Multivariate model adjusted for age, sex, marital status, equivalent income, educational level, smoking, alcohol consumption, job type, K6, number of the employee at the workplace, and cumulative incidence rate of COVID‐19 at the prefecture

## 4 DISCUSSION

Of the employees surveyed, 26% telecommuted at least 1 day a week. Of these, about 10% did not prefer to telecommute. Our data showed that the frequency of telecommuting more than once a week and the lack of preference for this way of working were associated with increased alcohol consumption. In addition, not preferring to telecommute but telecommuting at least once a week was significantly associated with increased alcohol consumption. By contrast, telecommuting was not associated with an increase in alcohol consumption when telecommuting was preferred. We are aware of no previous study that has examined the association between the desire to telecommute and increased alcohol consumption.

We found that telecommuters had increased alcohol consumption compared to employees who were not telecommuting. Previous studies have also reported that drinking increases among employees who telecommute and have fewer opportunities to go out. In a study of 3,000 telecommuters in the United States, 32% were more likely to drink during work hours than when they were in their regular workplaces.^13^ Another study found that 18% of Canadians who telecommuted and were less likely to go out drank more frequently.^14^ The causes of increased drinking among telecommuters include social isolation, loneliness, a poor work environment, and disrupted lifestyle.^13^, ^14^ In response to these stressors, employees start to drink more as a coping behavior. In addition, without the inhibitions from monitoring by co‐workers and superiors, it is easy to increase alcohol consumption. For this reason, the amount of alcohol consumed by telecommuters is likely to increase. In this study, working from home when this was not the preferred practice was associated with increased alcohol consumption.

In contrast, when workers desired to telecommute, telecommuting was not associated with increased alcohol consumption. The results suggest that a mismatch between attitude toward telecommuting and actual working practice, rather than telecommuting itself, is associated with increased alcohol consumption. Whether employees prefer to telecommute depends on which of the merits and demerits of telecommuting has a stronger impact on them. For example, workers who commute long hours may prefer to work from home, while those with no suitable working space at home may prefer not to telecommute. If demerits are perceived more strongly, then telecommuting will not be preferred. Reasons for not wanting to telecommute, such as social isolation, loneliness, poor work environment, and difficulty in establishing a daily rhythm, are consistent with those reported to lead to increased alcohol consumption in previous studies. Drinking alcohol may be a coping behavior against psychological distress caused by social isolation, loneliness, poor work environment, and disordered life rhythm.^15^

The mismatch between preferred and actual telecommuting practices may be a risk factor for inappropriate drinking behavior. It has been pointed out that telecommuting can lead to inadequate on‐site supervision,^9^ not only with regard to safety but also the management of employee health. It is difficult for companies to supervise the private life of individual employees, especially their lifestyle. However, inappropriate drinking is associated with health problems including alcohol dependence and liver dysfunction,^16^ along with other problems including presenteeism, decreased productivity, and accident risks.^17^, ^18^, ^19^, ^20^, ^21^ Therefore, deterrence programs and early detection of inappropriate drinking are important issues in occupational health. As telecommuting is expected to become a more widespread and long‐term way of working in many companies, placement of employees in ways that are consistent with their preferences for work locations is an important issue.

Several limitations of this study warrant mention. First, because we conducted an internet monitoring survey, the general applicability of the results is uncertain. However, we sought to minimize any bias in the participants by sampling across regions, occupations, and prefectures according to the incidence of infection. Second, we relied on self‐reported assessments of alcohol consumption. In general, it has been shown that drinkers tend to underestimate their consumption,^22^ which means that the amount of alcohol consumed might also have been underreported in this study. However, given the anonymous nature of the online survey, we believe that underreporting was relatively unlikely. Third, because this is a cross‐sectional study, the temporal relationship and direction of causality between telecommuting and increased alcohol consumption remains unknown. Fourth, the amount of alcohol consumption was assessed only as to whether or not it had increased, and the detailed change in the amount was unknown.

Under the COVID‐19 pandemic, telecommuting is recommended as an infection prevention measure. In many cases this has led to the introduction of telecommuting regardless of the preference of the employee, resulting in a mismatch. In this study, 10% of telecommuters did not prefer telecommuting to regular commuting to the workplace. Telecommuting despite no preference for it was significantly associated with increased alcohol consumption. The mismatch between telecommuting‐related preferences and imposed practices may be a new risk factor for increasing alcohol consumption, and therefore an emerging issue in occupational health.

## DISCLOSURE

*Approval of the research protocol*: the research was approved by the Ethics Committee of the University of Occupational and Environmental Health, Japan (Reference No. R2‐079). *Informed Consent*: informed consent was obtained via a form on the website. *Registry and the Registration No. of the study/trial*: N/A. *Animal Studies*: N/A. *Conflict of Interest*: None declared.

## AUTHORS' CONTRIBUTION

CW, YK, and YF conceived the research idea; AH, MN, KM, ST, MT, AO, and TN collected the data; YK, RY, and YF analyzed the data; CW led the writing of the manuscript. All authors participated in critically reviewing the study.
